# Two Cases of Lung Nodules Presenting With Kappa Light Chain Deposition Disease/Congo‐Red Negative AL Amyloidosis

**DOI:** 10.1155/crip/1819457

**Published:** 2026-11-09

**Authors:** Saltanat Ualiyeva, Atreyee Basu

**Affiliations:** ^1^ Department of Pathology and Laboratory Medicine Tufts Medical Center Boston Massachusetts USA tuftsmedicalcenter.org

**Keywords:** lung pathology, monoclonal immunoglobulin light chains, nonamyloid deposits, pulmonary light chain deposition disease

## Abstract

Light Chain Deposition Disease (LCDD) is a rare disorder characterized by the extracellular accumulation of monoclonal immunoglobulin light chains in various organs, most commonly the kidneys, but occasionally the heart, liver, and lungs. Pulmonary LCDD (PLCDD) is an exceedingly rare manifestation that can closely resemble other pulmonary diseases both radiologically and histologically. Unlike amyloidosis, LCDD does not exhibit Congo red positivity or apple‐green birefringence under polarized light, which can complicate diagnosis. Mass spectrometry (MS) has emerged as a valuable diagnostic tool in such cases, providing definitive identification of monoclonal light chains when traditional histopathological techniques are inconclusive. We present two cases of pulmonary nodular LCDD, both in women with autoimmune backgrounds, including Sjögren′s syndrome. Both patients presented with pulmonary nodules demonstrating FDG uptake on PET‐CT scans. Histologic examination revealed amorphous eosinophilic deposits without Congo red birefringence. Proteomic analysis via laser microdissection and liquid chromatography–mass spectrometry (LC‐MS) confirmed the presence of kappa light chain deposits, supporting a diagnosis of LCDD or non‐Congophilic AL‐type amyloidosis. One patient had no evidence of systemic disease, whereas the other showed clonal plasma cell infiltration with kappa light chain restriction, suggestive of a low‐grade B‐cell neoplasm. These cases underscore the diagnostic complexity of PLCDD and highlight the critical role of MS in differentiating it from pulmonary classical amyloidosis. Nodular PLCDD may follow an indolent course and often lacks systemic involvement, whereas diffuse forms carry a poorer prognosis because of extensive pulmonary and systemic involvement. Treatment remains focused on managing the underlying plasma cell disorder, though standardized therapeutic protocols are lacking because of the rarity of the condition. Our findings emphasize the need for increased clinical awareness and advanced diagnostic strategies to appropriately identify and manage PLCDD.

## 1. Introduction

Light Chain Deposition Disease (LCDD) is a rare and complex form of systemic extracellular deposition of monoclonal immunoglobulin light chains in organs like kidneys, heart, and liver [1–4]. It is a type of monoclonal gammopathy where the light chains produced by plasma cells misfold, aggregate, and deposit in tissues, leading to organ dysfunction [5]. LCDD is distinct from other forms of amyloidosis because of the absence of amyloid fibrils typically seen in light chain amyloidosis (AL), yet the clinical presentation and organ involvement often overlap [6].

Histologically, LCDD can be challenging to differentiate from other forms of deposition diseases, including AL amyloidosis and systemic light chain amyloidosis, because of the similarities in the pattern of organ involvement. However, LCDD is unique in that it does not show the Congo red positivity seen in traditional amyloidosis [7] and it lacks the characteristic birefringence under polarized light typically associated with amyloid deposits. Diagnosis requires a combination of clinical evaluation, immunohistochemical staining, and proteomic analysis, including mass spectrometry, to identify the monoclonal light chains responsible for the deposition.

Although treatment of LCDD remains largely focused on addressing the underlying plasma cell disorder [8], the prognosis depends heavily on the degree of organ damage at the time of diagnosis [9, 10]. Early recognition and intervention are critical in preventing irreversible organ dysfunction.

LCDD rarely affects lung and only sporadic cases of PLCDD have been described [11], usually associated with an underlying plasma cell dyscrasia or autoimmune process. The exact pathophysiology of pulmonary involvement in LCDD remains poorly understood but is thought to involve the direct deposition of monoclonal light chains in the pulmonary interstitium, alveolar walls, or vasculature. These deposits can cause local inflammation, fibrosis, and subsequent disruption of normal lung architecture [12–14]. In some cases, this deposition can mimic other forms of pulmonary disease, such as pulmonary fibrosis or interstitial lung disease [15].

Radiologically, PLCDD may present as diffuse interstitial infiltrates, pleural effusions, or nodular opacities on chest imaging, though these findings are nonspecific and can overlap with other diseases [16, 17]. Histopathologic examination of lung tissue typically reveals monoclonal light chain deposits within the alveolar walls or interstitial spaces, often identified by immunohistochemistry, and negativity on Congo red staining is used to differentiate from amyloid deposition [14].

Histologically, PLCDD has nodular and diffuse forms [12]. Nodular PLCDD is less common and typically presents as solitary or multiple pulmonary nodules [15]. These nodules are often discovered incidentally during imaging for unrelated conditions. Radiologically, they may mimic neoplastic processes such as metastatic disease or primary lung cancer, necessitating histological confirmation [14]. Nodular lesions in PLCDD consist of localized deposits of monoclonal light chains, usually without associated inflammation or significant fibrosis.

On histology, these nodules are composed of eosinophilic deposits that stain positively for light chains (*κ* or *λ*) by immunohistochemistry. Unlike amyloid, these deposits are Congo red‐negative and do not show apple‐green birefringence under polarized light. The nodular form may represent an indolent or localized disease, and in some cases, no systemic involvement is found, leading to the term localized PLCDD.

In PLCCD, Masson trichrome stain shows the deposited material as positive red (or crimson red) rather than gray‐purple and dark green hues that is typical for amyloid deposits [18]. The red reaction suggests that the material is noncollagenous (i.e. not fibrosis) and is proteinaceous deposition (light chains).

In contrast, the diffuse form of PLCDD is more aggressive and typically presents with progressive respiratory symptoms such as dyspnea, cough, and hypoxia. Imaging often reveals a reticulonodular or ground‐glass interstitial pattern, resembling other interstitial lung diseases [19]. The diffuse pattern reflects widespread deposition of light chains along alveolar basement membranes, interstitium, and vasculature, often leading to interstitial fibrosis and restrictive lung disease [20].

Histopathologically, the diffuse form shows extensive infiltration of the interstitium with light chain deposits, sometimes accompanied by interstitial fibrosis and chronic inflammatory infiltrates [20]. This form is often associated with systemic plasma cell dyscrasias such as multiple myeloma, and the prognosis tends to be poorer compared with the nodular type because of the extent of pulmonary involvement and underlying systemic disease.

In terms of prognosis, pulmonary involvement in LCDD is often associated with a poor outcome, primarily because of the progressive nature of the disease and its association with underlying hematologic malignancies, such as multiple myeloma or Waldenström′s macroglobulinemia [11]. Early diagnosis and treatment are essential for preventing further pulmonary damage, although the therapeutic options for pulmonary LCDD remain limited, focusing primarily on managing the underlying plasma cell dyscrasia and supportive care.

Here we describe two patients with pulmonary LCDD presenting as lung nodules with LCDD/non‐Congophilic AL amyloidosis.

## 2. Case presentation

### 2.1. Case No. 1

The first case is a 66‐year‐old female who presented with emphysema with dyspnea on exertion and multiple lung nodules, which have been stable on computer tomography (CT) since 2013. She was also found to have a positive ANA with a high titer 1:5120. Rheumatology diagnosed Sjögren′s syndrome with patient having positive SSA (anti‐Ro) and SSB (anti‐La) antibodies.

On her recent chest CT, she had a new anterior right upper lobe lung nodule, which had fluorodeoxyglucose (FDG) with a standardized uptake value (SUV) = 3.7. Within 3 years, the right upper lobe nodule demonstrated growth from 1.9 × 0.5 × 1.5 cm to 2.4 × 1.3 × 1.7 cm. This nodule with intense FDG uptake had eccentric calcification, suspicious for indolent primary bronchogenic neoplasm.

The patient underwent a robotic right upper lobe wedge resection. Microscopic examination revealed a pulmonary nodule (2.5 × 2.0 × 2.0 cm), consisting of pink amorphous eosinophilic material, histomorphologically suggestive of amyloidosis (Figure 1A,B). However, Congo red special stain (Figure 1C) did not show apple‐green birefringence in this nodule and special stain for trichrome showed bright red color in the material (Figure 1D).

**Figure 1:**
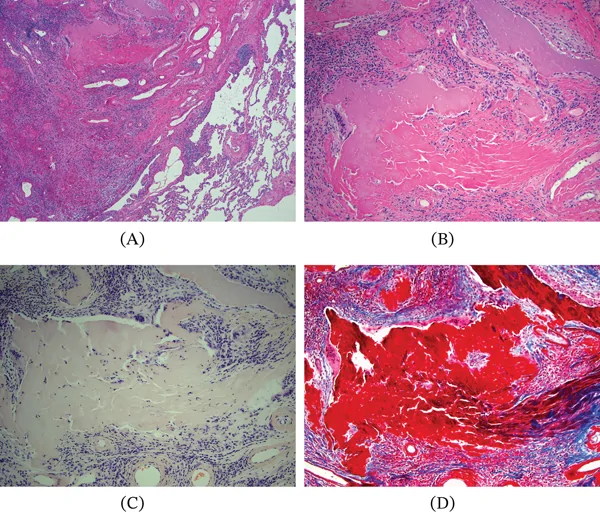
(A and B): H&E stain shows lung parenchyma with eosinophilic amorphous material (A‐40x, B‐200x). (C): Congo‐red special stain without polarization (200x). (D): Trichrome stain (200x).

Liquid‐chromatography–mass‐spectrometry (LCMS) (Mayo Clinical Laboratories) on paraffin‐embedded blocks did not identify proteomic features of amyloid. The specimen contained a predominance of peptides associated with kappa immunoglobulin light chains, consistent with kappa LCDD.

Histologically, there was background lymphoplasmacytic inflammation along with foreign body type giant cell reaction and organization. Kappa and Lambda in situ hybridization demonstrated that the plasmocytic infiltrate is kappa‐restricted. Kappa‐to‐Lambda expression was 15:1. The findings indicated involvement by plasma cell neoplasm. The nodule also revealed focal osseous metaplasia and calcification. The overall findings were consistent with mature B cell lymphoma with plasmocytic differentiation and kappa light chain restriction.

Serum protein electrophoresis (SPEP) and immunofixation electrophoresis (IFE) showed polyclonal increase in gamma globulins with no abnormal bands. Urine protein electrophoresis (UPEP) had no significant protein in the urine, but urine IFE showed a faint questionable band in the kappa immunoglobulin lane, at the limit of visual detection, migrating electrophoretically in the beta globulin region of the gel. Total protein level was normal at 8.5 g/dL with albumin being within normal limits at 4.3 g/dL. Free kappa chains were slightly increased at 67 mg/L (normal range 3.3–19.4 mg/L), whereas free lambda chains were not significantly increased.

Given the patient was asymptomatic with no pulmonary symptoms and her recent PET/CT no additional FDG avid lesions, no treatment was recommended. The patient has been followed by rheumatology and pulmonology clinics.

### 2.2. Case No. 2

The second case is a 57‐year‐old woman with Sjögren′s syndrome who presented with shortness of breath, concerning for interstitial‐lung‐disease. She underwent chest CT which revealed a 1.7 cm lung nodule in the right upper lobe. On repeat scanning, the nodule was increasing in size and PET showed metabolic activity with SUV of 7.3.

The lesion was biopsied and histologically examined. Tissue analysis revealed lung parenchyma with benign bronchial epithelium, amorphous material and multinucleated giant cell reaction (Figure 2A,B). The amorphous material was morphologically suggestive of amyloid, although Congo red stain (Figure 2C) did not show the expected apple‐green birefringence. Trichrome stain highlights focal bright red color in the material deposited in the lung parenchyma (Figure 2D).

**Figure 2:**
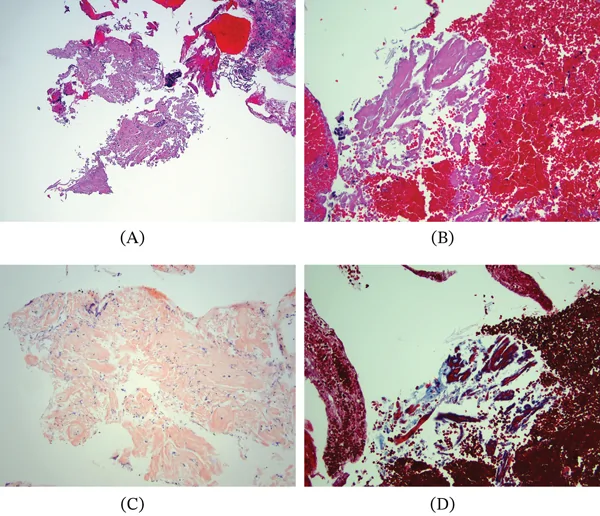
(A and B): H&E stain shows lung parenchyma with eosinophilic amorphous material (A‐40x, B‐200x). (C): Congo‐red special stain without polarization (200x). (D): Trichrome stain (200x).

LCMS (Mayo Clinical Laboratories) was performed on peptides extracted from amorphous extracellular material from micro dissected areas of the paraffin‐embedded specimen. LCMS detected predominance of peptides associated with kappa immunoglobulin light chains. It also detected amyloid chaperone proteins from non‐Congophilic material.

Hematopathology evaluation revealed no evidence of a lymphoproliferative disorder.

SPEP and IFE have not revealed any abnormal protein. Urine IFE showed a faint noncomigrating band in the lambda immunoglobulin lane in a background of polyclonal immunoglobulins. Total protein level was slightly increased at 9.7 g/dL with albumin being within normal limits at 3.9 g/dL. Free kappa chains were increased at 80 mg/L (normal range 3.3–19.4 mg/L), and free lambda chains were within normal limits.

The overall findings support an immunoglobulin‐associated process and are between that of kappa LCDD and AL (kappa)‐type amyloidosis.

The patient has been regularly monitored. The right upper lobe nodule remains unchanged. Currently the patient does not have any demonstrative evidence of end organ damage pertaining to her kidneys, cardiac function, and recent cardiac testing was without evidence of coronary arterial disease or significant hypertrophy.

## 3. Discussion

In the lungs, LCDD may lead to a range of respiratory symptoms, including cough, dyspnea, pleuritic chest pain, and, in more severe cases, respiratory failure [12]. PLCDD typically presents radiologically with multiple nodules and cysts, sometimes accompanied by ground‐glass opacities or interstitial changes, often with increased FDG uptake on PET. FDG uptake in PLCDD lesions has been attributed to inflammation and fibrosis around light‐chain deposits rather than malignant transformation [21].

Both LCDD and amyloidosis may rarely involve the lung resulting in the deposition of abnormal proteins. LCDD involves the deposition of monoclonal immunoglobulin light chains, commonly seen in Sjögren′s syndrome [22], whereas amyloidosis involves the deposition of Congophilic amyloid fibrils.

Mass spectrometry has emerged as a valuable tool in diagnosing LCDD, particularly in cases where traditional methods like Congo red staining and immunofluorescence yield inconclusive results [23]. By identifying specific immunoglobulin light chain peptides in tissue deposits, MS enables precise characterization of the disease, aiding in distinguishing it from other conditions such as amyloidosis. MS offers high sensitivity and specificity in identifying low‐abundance proteins and provides detailed information on the amino acid sequence of deposited proteins, allowing for precise identification of the immunoglobulin subtype involved.

Treatment of PLCDD is challenging because of its rarity and the absence of standardized guidelines. However, emerging evidence suggests that therapies effective in systemic LCDD, such as autologous stem cell transplant and bortezomib‐based regimens, may also benefit patients with isolated pulmonary involvement [10, 24, 25]. Individual responses to therapy can vary, and some patients may require adjustments in treatment regimens. Ongoing monitoring is essential to assess treatment efficacy and detect potential relapses or complications.

In summary, we report two extremely rare cases of PLCDD in patients with underlying autoimmune diseases. Microscopically, the nodules consisted of amorphous eosinophilic deposits, suggestive of amyloid; however, they were not birefringent on Congo red stains. Mass spectrometry confirmed presence of light chains, consistent with diagnosis of LCDD. One of the patients was diagnosed with mature B cell lymphoma with plasmocytic differentiation and kappa light chain restriction. The other patient did not have hematological malignancy. Fortunately, both patients have been stable and monitored in clinics.

## Funding

No funding was received for this manuscript.

## Conflicts of Interest

The authors declare no conflicts of interest.

## Supporting information


**Supporting Information** Additional supporting information can be found online in the Supporting Information section. Supporting Information. Data S1: CARE checklist of information to include when writing a case report.

## Data Availability

The data that support the findings of this study are available from the corresponding author upon reasonable request.
