# Supplementary material for: Two Cases of Lung Nodules Presenting With Kappa Light Chain Deposition Disease/Congo‐Red Negative AL Amyloidosis
Source: Case Rep Pathol. 2026 Nov 9;2026:1819457. doi: 10.1155/crip/1819457 (PMC13748704; doi:10.1155/crip/1819457)
Supplement: Supplementary file 1 [file CRIP-2026-1819457-s001.pdf]

CARE case report guidelines

CARE Checklist of information to include when writing a case report

(cc) BY-NC-ND

Topic	Item	Checklist item description	Reported on Line
Title	1	The diagnosis or intervention of primary focus followed by the words "case report"	104
Key Words	2	2 to 5 key words that identify diagnoses or interventions in this case report, including "case report"	35
Abstract (no references)	3a	Introduction: What is unique about this case and what does it add to the scientific literature?	20
	3b	Main symptoms and/or important clinical findings	18
	3c	The main diagnoses, therapeutic interventions, and outcomes	21
	3d	Conclusion—What is the main "take-away" lesson(s) from this case?	33
Introduction	4	One or two paragraphs summarizing why this case is unique (**may include references**)	39
Patient Information	5a	De-identified patient specific information.	108
	5b	Primary concerns and symptoms of the patient.	108
	5c	Medical, family, and psycho-social history including relevant genetic information	108
	5d	Relevant past interventions with outcomes	111
Clinical Findings	6	Describe significant physical examination (PE) and important clinical findings.	108
Timeline	7	Historical and current information from this episode of care organized as a timeline	110
Diagnostic Assessment	8a	Diagnostic testing (such as PE, laboratory testing, imaging, surveys).	113
	8b	Diagnostic challenges (such as access to testing, financial, or cultural)	120
	8c	Diagnosis (including other diagnoses considered)	133
	8d	Prognosis (such as staging in oncology) where applicable	147
Therapeutic Intervention	9a	Types of therapeutic intervention (such as pharmacologic, surgical, preventive, self-care)	147
	9b	Administration of therapeutic intervention (such as dosage, strength, duration)	147
	9c	Changes in therapeutic intervention (with rationale)	147
Follow-up and Outcomes	10a	Clinician and patient-assessed outcomes (if available)	148
	10b	Important follow-up diagnostic and other test results	129
	10c	Intervention adherence and tolerability (How was this assessed?)	131
	10d	Adverse and unanticipated events	205
Discussion	11a	A scientific discussion of the strengths AND limitations associated with this case report	212
	11b	Discussion of the relevant medical literature **with references**.	184
	11c	The scientific rationale for any conclusions (including assessment of possible causes)	190
	11d	The primary "take-away" lessons of this case report (without references) in a one paragraph conclusion	209
Patient Perspective	12	The patient should share their perspective in one to two paragraphs on the treatment(s) they received	N/A
Informed Consent	13	Did the patient give informed consent? Please provide if requested	Yes ☐ No ☑
